# COVID-19 vaccine hesitancy among adults in Hawassa City Administration, Sidama Region, Ethiopia: A community-based study

**DOI:** 10.3389/fpubh.2023.1122418

**Published:** 2023-03-02

**Authors:** Seblewongel Yohannes, Akalewold Alemayehu, Yohannes Markos Woldesenbet, Temesgen Tadele, Desta Dangiso, Muntasha Birhanu, Endrias Markos Woldesemayat

**Affiliations:** ^1^Sidama Region Health Department, Hawassa, Ethiopia; ^2^School of Public Health, College of Medicine and Health Science, Hawassa University, Hawassa, Ethiopia; ^3^Assistant Professor of Medical Physiology, Department of Biomedical Sciences, Faculty of Medical Sciences, Institute of Health, Jimma University, Jimma, Ethiopia

**Keywords:** COVID-19, vaccine acceptance, Hawassa City, Tula, adults

## Abstract

**Objective:**

The COVID-19 vaccine is one of the key measures to control the disease. However, some people are hesitant to take the vaccine. The objective of this study was to assess COVID-19 vaccine hesitancy and associated factors among adults in Hawassa City Administration, South Ethiopia.

**Method:**

From March 1 to 30, 2022, we conducted a community-based cross-sectional study among randomly selected 622 adults in Hawassa City Administration. A multi-stage sampling technique was used to recruit the study participants. Data were collected through a pretested structured questionnaire that was administered by four trained high school graduates. Data entry and analysis were done using the SPSS version 20 statistical package. Descriptive statistics and logistic regression analysis were performed. Statistically significant associations were reported at *p*-value <0.05.

**Result:**

Among the participants, 400 of them (64.3%) had a high level of knowledge about the COVID-19 vaccine) and 425 of them (68.3%) had a positive attitude toward the COVID-19 vaccine. The level of vaccine hesitancy was 165 (26.5%) and vaccine acceptance was 457 (73.5%). The main reason for willingness to take the vaccine was to protect oneself from COVID-19 (364 participants, 58.5%), and for unwillingness, it was fear of the vaccine (154 participants, 24.8%). Mass media was the main source of information about the vaccine (472 participants, 75.9%). Age (adjusted odds ratio (AOR): 2.1, 95% CI: 1.2–3.7), religion (AOR: 2.6, 95% CI: 1.1–5.9), history of COVID-19 disease (AOR: 4.6, 95% CI: 1.4–14.9), knowledge related to the COVID-19 vaccine (AOR: 1.9, 95% CI: 1.2–3.1), and attitude toward the COVID-19 vaccine (AOR: 13.2, 95% CI: 8.3–20.9) were factors associated with vaccine hesitancy.

**Conclusion:**

A low proportion of COVID-19 vaccine hesitancy was observed among our study participants. Improving people's awareness could help to improve vaccine acceptance. It is important to focus interventions on the identified risk factors of vaccine hesitancy.

## Background

Since COVID-19 was declared a global pandemic by the World Health Organization (WHO), it spread across the world and caused high mortality and morbidity (1). The disease disrupted health systems in many countries (2). To date, there is no definite antiviral treatment for COVID-19 (3). However, promising COVID-19 vaccines were produced and are being used, necessitating consideration of their potential demand, distribution, and adoption to obtain their desired effect (4). COVID-19 control could depend on the vaccine and its successful delivery to a large portion of the population (5). Data on COVID-19 vaccination status shows 68% of the world population received at least one dose of a COVID-19 vaccine, while only 22.7% of people in low-income countries received at least a dose of the vaccine (6). Though governments are advocating vaccination, some anti-vaccination activists are campaigning against the need for a vaccine claiming the nonexistence of COVID-19 (7).

Ethiopia is among the five African countries most burdened by COVID-19 (8). On March 13, 2020, the first COVID-19 case was identified in the country (9). The COVID-19 updated data in Ethiopia shows that there were over 493,996 positive cases and 7,572 deaths in the country (10). The government has been working to disseminate information on COVID-19 prevention measures through mass media and other modes (11).

Globally, the reported rate of vaccine acceptance ranged from 23.6 to 97% with the least vaccine hesitancy at 76.4% (12). The vaccine hesitancy levels in two different settings in China were 19.4 and 11.7% (13). The COVID-19 vaccine hesitancy rate among the general population in Saudi Arabia was 55.3% (14) and 69.1% in the United Arab Emirates (15). Another study reported a 12.4% hesitancy rate toward COVID-19 vaccination (16). The COVID-19 vaccine acceptance rate in Addis Ababa, Ethiopia ranged from 33.3–69.8% with an average of 51.8% (17–19). In the Amhara region, Ethiopia, the estimate was 54.2% (95% CI of 39.4–68.3%) (20–22). A similar figure from studies in the southern region of Ethiopia placed the rate at 57.0% (95% CI of 44.4–68.7%) (23–25). Other studies from Southern Ethiopia (26) reported a proportion of 62.6% in the Gurage Zone and 45.5% in Sodo City, Wolaita Zone (27, 28). The pooled prevalence of COVID-19 vaccine acceptance in Ethiopia was 57.8% (95% CI: 47.2–67.8%) (29). Another study by Mose et al. reported a 51.64% pooled national prevalence of COVID-19 vaccine acceptance (30).

Factors associated with vaccine hesitancy included socio-demographic variables, such as gender, nationality, age group, living condition, education, and occupation, with men who were young and with a low educational level showing higher hesitancy rates. In cases of increased perceived personal or public risk of contracting the disease and a high perception of serious outcomes from the disease, there was an increased vaccine acceptance (14, 15, 28, 31, 32). Vaccine safety, side effects, the desire to have natural immunity, not receiving the influenza vaccine, perceived low risk of contracting the disease, and decreased perception of serious outcomes due to COVID-19 were factors associated with vaccination hesitancy (11, 15, 33). Other factors, such as being tested for COVID-19, family members or friends being diagnosed with COVID-19, systemic diseases, not being previously infected with COVID-19, following updates on the COVID-19 vaccine, having mental disorders and having a high-risk perception of COVID-19 infection were significantly associated with COVID-19 vaccine acceptance (28, 31, 32). Additional factors that showed association with vaccine acceptance included the belief that authorities were handling the pandemic adequately (15), confidence in government decisions, the feeling of responsibility to stop the pandemic, and receiving vaccinations in childhood (15, 28).

Various studies on COVID-19 vaccine hesitancy have been done in different settings in Ethiopia (29). However, to the best of our knowledge, no research has been conducted in the Sidama Region, which consists of approximately four million people. Knowing the level of COVID-19 hesitancy and associated factors in different settings is important. The result of the current study will increase our evidence on the problem so that there can be public health action undertaken in the study area. Therefore, in this study, we aimed at filling the gap in assessing the level of the hesitancy of COVID-19 vaccination and factors contributing to vaccine hesitancy among adults in the Hawassa City Administration, Sidama Region, South Ethiopia.

## Methods and materials

### Study design, setting, and period

The study design used in this research was a community-based cross-sectional study. It was conducted in Hawassa City Administration, Sidama region, South Ethiopia. Hawassa is the capital of the Sidama and Southern Nations, Nationalities, and People's Regions in Ethiopia. The city is divided into eight sub-cities with 32 Kebeles (20 urban and 12 rural). The City Administration is located 275 km south of Addis Ababa, the capital of Ethiopia. In 2018 the total population of the city was estimated to be 374,034 (34). The city administration has eight hospitals (four public and four private) and 12 health centers that render health services to the population in the town. This study was conducted from March 1 to March 30, 2022.

### Population

All adult populations over the age of 18 years were included in the source population. Adults over the age of 18 years who lived in the study kebeles (smallest administrative unit) for at least six months were selected as study participants from the source population. Respondents who were mentally or seriously ill at the time of data collection and those not willing to participate in the study were excluded from the study.

### Sample size and sampling procedures

The sample size was calculated by using the single population proportion formula. A prevalence of 45.5% COVID-19 vaccination acceptance in the Wolaita Zone, Southern Ethiopia was considered (28). Using a 95% confidence interval (CI), 5% of marginal error, 10% non-response rate, and 1.5 design effect, the minimum sample size estimated for the study was 627. The study participants were recruited using a multi-stage sampling technique. First, a purposive sampling technique was used to select a rural Kifleketema (Tula sub-city) and random sampling to select three out of seven urban sub-cities; namely Tabor, Menaheria, and Gebeyadar sub-cities. Then four out of 12 kebeles were recruited from the rural sub-city and four out of 20 kebeles were recruited from the urban sub-cities. Then a proportional number of people from each kebele was randomly selected. Households were selected by a systematic random sampling technique and only one eligible individual was interviewed from the selected households. The first household was randomly selected from the kebele and then every tenth household was selected to identify the study participant. When two or more individuals in a household were eligible, we selected one by a simple random sampling technique. The total number of people living in the selected kebeles was 111,640. The estimated number of eligible people living in the selected kebeles was 53,000.

### Variables of the study

The dependent variable considered in the study was COVID-19 vaccine hesitancy. Independent variables that were measured in the study were socio-demographic characteristics, such as age, sex, ethnicity, marital status, address, religion, occupation, level of education, and income. Knowledge of the COVID-19 vaccine, attitude toward the COVID-19 vaccine, vaccine-related factors, COVID-19 infection-related characteristics, other morbidities, and communication-related factors were other independent variables measured in the study.

### Data collection tools and procedure

A pretested and interviewer-administered questionnaire was used to collect the data. The instrument was developed after an analysis of previous studies in the area. The following factors were included in the questionnaire: socio-demographic variables, such as age, sex, occupation, and marital status; attitude toward COVID-19 as stated below in the operational definitions section; knowledge of COVID-19 related variables, which consisted of seven COVID-19 knowledge-related variables, individual health-related variables such as having chronic diseases, having had COVID-19, media using, childhood vaccination, family, friends and relatives having a history of COVID-19 sickness; willingness to receive COVID-19 vaccine and the reasons of willingness or unwillingness to take the vaccine. The responses to questions assessing variables' attitudes toward COVID-19 and knowledge of COVID-19 were classified in agree, neutral, and disagree. For a positive question, participants who responded agree were considered as having a correct response while other responses were considered incorrect. Details on the responses to each question used in assessing the variables' attitude toward COVID-19, knowledge of COVID-19, and willingness to receive the COVID-19 vaccine were described in the operational definition.

The questionnaire was first prepared in English and then it was translated into Amharic and then it was translated back to English to check for consistency in meaning. Four high school graduates were recruited from Hawassa city to act as enumerators. Enumerators were trained about the objectives of the study and how to administer the questionnaire. Enumerators collected the data through face-to-face interviews by identifying participants from the communities.

### Data quality control

Data collectors and supervisors were trained on issues related to the research aim, data collection methods, and data collection tools. We did a pretest on 5% of the sample size among adults in one of the kebele located adjacent to the sampled kebeles. These cases were not included in the main study. Data were collected by four trained high school graduates through a pretested structured questionnaire. The data collection process was supervised by the principal investigator. If participants failed to provide a response to a question, they were not allowed to continue to the next question. Collected data were checked daily for completeness and clarity. Necessary feedback was offered to data collectors before the next day of data collection began.

### Operational definitions

COVID-19 vaccine acceptance: COVID-19 vaccine acceptance was measured by asking a yes or no response question on willingness to take the vaccine; are you willing to take the COVID-19 vaccine?

Attitude toward COVID-19 vaccine: was measured by asking nine COVID-19 vaccine attitude-related questions. These questions referred to worries about the COVID-19 vaccine, worries about vaccine use, whether the participant preferred the body's natural defense over the vaccine, whether the participant thought that without the vaccine the disease could not reduce, the participant's willingness to take the vaccine if it was available and delivered, the participant's attitude in using other methods of prevention to prevent COVID-19 other than the vaccine, the participant's belief of the vaccine effectiveness, the participant's beliefs that the vaccine has no side effects, and the participant's belief about the vaccine preventing complications due to COVID-19 disease. Participants who had four to nine positive responses were considered as having a positive attitude toward the vaccine and otherwise, they were considered as having a negative attitude.

Knowledge of the COVID-19 vaccine: was measured by asking seven COVID-19 vaccine knowledge-related questions. Having a response of at least four positive answers was considered as having a good knowledge of the COVID-19 vaccine. For this assessment, the questions referred to the participant's knowledge about the treatment for COVID-19, knowledge of the existence of the vaccine, the vaccine's effectiveness, whether the participant thought unvaccinated people could contract the disease, the specific method of COVID-19 prevention, how many vaccine doses are required to be fully vaccinated and the side effects related to the vaccine.

### Data analysis

Data were entered into the SPSS version 20 statistical software. Descriptive statistics were reported. Both bivariate and multivariable logistic regression models were used to identify associated factors of vaccine acceptance. We used a two-sided test to prove our hypothesis. We performed a reliability analysis to test the internal consistency of responses by using Cronbach's Alpha test for the variables' attitude toward COVID-19 and knowledge of COVID-19. The test showed good alignment. Variables with a p-value of less than or equal to 0.2 in the bivariate logistic regression model were entered into the multivariable logistic regression model to control the effect of confounding variables. The variables included in the model were age, occupation, religion, chronic diseases, COVID-19 disease, whether the participant had friends with COVID-19, contact with COVID-19 cases, knowledge assessment, and attitude assessment. Odds ratios and their 95% confidence intervals (CI) were computed and variables with a p-value less than 0.05 were considered for reporting a statistically significant association.

### Ethical approval

Ethical clearance was obtained from the institutional review board of Hawassa University, College of Medicine and Health Sciences Reference number IRB/129/14. Letter of permission was obtained from responsible offices found at various levels; the School of Public Health, Hawassa City Administration Health Department, and the selected Kebeles. Participants were informed about the purpose, benefits, risks, and the confidentiality of information collected. Participation in the study was on a voluntary basis, and all information was kept anonymous and confidential. Participants did not receive any gift or monetary compensation for being part of the study. Informed verbal consent was obtained from each study participant prior to the interview. Authors had no access to information that could identify individual participants during or after data collection.

## Results

Among 627 eligible respondents, 622 people participated in the study, with a response rate of 99.2%. Five eligible individuals stopped the interview after beginning the interview process, so their questionnaires were excluded from the analysis. Over half, 330 (53.1%), of the study participants were between the age of 18–29 years and 320 (51.4%) were male. Employed participants constituted 379 (60.9%) and 291 (46.8%) participants had above the secondary level of education. Most of the participants were married, 395 (63.5%) and protestant 402 (64.6%) (Table 1).

**Table 1:** Socio-demographic characteristics of the study participants.

**Characteristics**	**Values**	**Number**	**%**
Age, median (IQR)	29 (23, 36)		
Age group in years	18, 29	330	53.1
	>29	292	46.9
Sex	Male	320	51.4
	Female	302	48.6
Occupation	Employed	379	60.9
	Unemployed	94	15.1
	Student	149	24.0
Education	No education	204	32.8
	Primary to secondary	127	20.4
	Above secondary	291	46.8
Marital status	Married	395	63.5
	Single	214	34.4
	Others	13	2.1
Religion	Protestant	402	64.6
	Orthodox	142	22.8
	Others	78	12.5
Income	<6,800	178	28.6
	>6,800	179	28.8
	Missing	265	42.6

COVID-19, coronavirus disease; IQR, interquartile range.

Of the participants, 38 (6.1%) had chronic diseases and 23 (3.7%) were diseased by COVID-19. The majority of the participants, 567 (91.2%), used media to access information. A high proportion of the study participants, 459 (73.8%), had taken their childhood vaccinations. Concerning the family history of COVID-19, 38 (6.1%) had a family history of COVID-19 disease, while 45 (7.2%) had relatives with history of COVID-19 illnesses. Only 89 (14.3%) of the study participants had friends with history of COVID-19 disease. Concerning COVID-19 testing, 268 (43.1%) were tested, and of these, 17 (2.7%) had positive test results. The majority of the study participants, 400 (64.3%), had good knowledge of the COVID-19 vaccine. Attitude toward the COVID-19 vaccine was positive for 425 (68.5%) of the study participants. The COVID-19 vaccine acceptance rate among the study participants was 457 (73.5%) (Table 2).

**Table 2:** Health and health-related characteristics of the study participants.

**Characteristics**	**Values**	**Number**	**%**
Chronic diseases	Yes	38	6.1
	No	584	93.9
Contracted COVID-19	Yes	23	3.7
	No	599	96.3
Media user	Yes	567	91.2
	No	55	8.8
Childhood vaccination	Yes	459	73.8
	No	125	20.1
	Don't know	38	6.1
Family history of COVID-19	Yes	38	6.1
	No	584	93.9
Relatives' history of COVID-19 sickness	Yes	45	7.2
	No	577	92.8
Friends' history of COVID-19 sickness	Yes	89	14.3
	No	533	85.7
COVID-19 cases contact history	Yes	138	22.2
	No	484	77.8
COVID-19 testing	Yes	268	43.1
	No	354	56.9
COVID-19 test result	Positive	17	2.7
	Negative	221	35.5
	Don't know	30	4.8
COVID-19 vaccine knowledge	Good	400	64.3
	Poor	222	35.7
COVID-19 vaccine attitude	Positive	425	68.3
	Negative	197	31.7
Vaccine acceptance	Yes	457	73.5
	No	165	26.5

COVID-19, coronavirus disease.

The first four reasons reported for willingness to take the vaccine were: to protect oneself, 364 (58.5%); to prevent family members or relatives from contracting COVID-19, 344 (55.3%); it was the advice of health professionals, 334 (53.7%); and thinking that the vaccine had no side effects, 201 (32.3%). The main reasons for unwillingness to take the vaccine were: fear of the vaccine, 154 (24.8%); preference for other prevention methods other than the vaccine, 140 (22.5%); being worried about the vaccine, 117 (18.8%); and fear of injections, 61 (9.8%). Out of the participants, 22 (3.5%) were unwilling to take the vaccine thinking that the vaccine caused COVID-19 (Table 3). Concerning sources of information about the COVID-19 vaccine, 472 (75.9%) obtained information from mass media, 430 (69.1%) of the participants said that they read about it on social media, 383 (61.6%) of the participants reported that they got information from health professionals, and 275 (44.2%) reported that they heard about it in campaigns.

**Table 3:** Reasons for willingness and unwillingness to take the COVID-19 vaccine in Hawassa City.

**Characteristics**	**Number**	**%**
Willing to take the vaccine	457	73.5
Protect oneself	364	58.5
Prevent family or relatives from contracting COVID-19	344	55.3
Following advice from health professional	334	53.7
Vaccine has no side effect	201	32.3
Useful for travels	133	21.4
Authorities mandated	43	6.9
Relatives died	26	4.2
Other reasons	2	0.3
Unwilling to take the vaccine	165	26.5
Fear of the vaccine	154	24.8
Prefer other type of prevention	140	22.5
Worried about the vaccine	117	18.8
Fear of injections	61	9.8
Vaccine is not effective	56	9.0
Vaccine causes COVID-19	22	3.5
No answer	19	3.1
Previous side effects	10	1.6
Other reasons	16	2.6

COVID-19, coronavirus disease.

Factors affecting COVID-19 vaccine hesitancy are presented in Table 4. Age, occupation, religion, having contracted COVID-19, knowledge related to the COVID-19 vaccine, and attitude toward the COVID-19 vaccine were factors that showed an association with COVID-19 vaccine hesitancy in a bivariate logistic regression analysis. Except for occupation, all other variables maintained significance in predicting vaccine hesitancy in a multivariate analysis. Participants in the age group of 18–29 had an increased risk of vaccine hesitancy rate (adjusted odds ratio (AOR): 2.1, 95% CI; 1.2–3.7). Orthodox Christians had higher odds of vaccine hesitancy (AOR: 2.6, 95% CI; 1.1–5.9) than participants in the other religions categories. Also, participants with a history of COVID-19 illness were more likely to be hesitant than those who had not contracted the disease (AOR: 4.6, 95% CI; 1.4–14.9). The COVID-19 vaccine hesitancy rate was higher for participants with low knowledge about the COVID-19 vaccine than for their counterparts (AOR: 1.9, 95% CI: 1.2–3.1). Similarly, the vaccine hesitancy rate was higher for participants with negative attitude toward the COVID-19 vaccine than for their counterparts (AOR: 13.2, 95% CI: 8.3–20.9).

**Table 4:** Factors affecting COVID-19 vaccine hesitancy among adults in Hawassa City.

**Characteristics**	**Values**	**Hesitancy**		**COR (95% CI)**	**AOR (95% CI)**
		**Yes**	**No**		
Age in years	18–29	107	223	1.9 (1.3–2.8)	2.1 (1.2–3.7)
	>29	58	234		
Occupation	Employed	85	294	
	Unemployed	24	70	1.2 (0.7–2.0)	1.3 (0.7–2.6)
	Student	56	93	2.1 (1.4–3.1)	1.7 (0.9–3.2)
Religion	Protestant	93	309	1.4 (0.7–2.6)	1.1 (0.5–2.4)
	Orthodox	58	84	3.2 (1.6–6.2)	2.6 (1.1–5.9)
	Others	14	64
Chronic diseases	Yes	7	31	0.6 (0.3–1.4)	0.9 (0.3-2.6)
	No	158	426
Contracted COVID-19	Yes	13	10	3.8 (1.6–8.9)	4.6 (1.4–14.9)
	No	152	447
Friend contracted COVID-19	Yes	29	60	1.4 (0.9–2.3)	0.9 (0.4–1.9)
	No	136	397
Contact with COVID-19 case	Yes	42	96	1.3 (0.8–1.9)	1.3 (0.7–2.5)
	No	123	361
Knowledge assessment	Low	90	132	2.9 (2.0–4.3)	1.9 (1.2–3.1)
	Good	75	325
Attitude assessment	Negative	123	74	15.2 (9.9-23.3)	13.2 (8.3-20.9)
	Positive	42	383

COR, Crude Odds Ratio; AOR, Adjusted Odds Ratio; CI, Confidence Interval.

## Discussion

The majority of the current study participants used mass media to gain information. There was a high level of good knowledge of the COVID-19 vaccine and a positive attitude toward the COVID-19 vaccine among theparticipants. Vaccine hesitancy among the study participants was low. The main reason for willingness of taking the vaccine was protection against the disease and the main reason for unwillingness was fear of the vaccine. Health professionals were the primary source of information about the vaccine. Age, religion, history of COVID-19 disease, knowledge related to the COVID-19 vaccine, and attitude toward the COVID-19 vaccine predicted vaccine hesitancy.

The current study finding of a vaccine acceptance rate of 73.5%, was relatively similar to the report from South East Asia, which observed a 71% acceptance rate (35). However, it was higher than the acceptance rates reported in the United Arab Emirates, 60.1% (15), in Saudi Arabia, 44.7% (14), in Wolayta Sodo, Ethiopia, 45.5% (28), in Addis Ababa, Ethiopia, 51.8% (17–19), in the Amhara region, 54.2% (20–22), in the South Ethiopia region, 57.0% (23–25), and the pooled prevalence reports of COVID-19 vaccine acceptance in Ethiopia, 57.8 and 51.6% (29, 30). However, it was lower than the study findings in other settings, such as Ecuador (97.0%), Malaysia (94.3%), Indonesia (93.3%), and China (91.3%) (12). The difference in the prevalence of vaccine acceptance could be related to factors such as differences in socio-demographic factors, socio-economical factors, and the difference in the time of assessment. Since fear of the vaccine was the primary reason for unwillingness to have it, as suggested by Di Giuseppe et al. (36), providing health education to avoid the fear of the vaccine could be an important measure to improve vaccine acceptance.

Data from the UK indicated that a critical factor for vaccine hesitancy was anxiety (37). Similarly, in the current study, the main reason for unwillingness to take the COVID-19 vaccine was fear of the vaccine. There were misconceptions about willingness in the current study. A significant proportion of the study participants reported that they were willing to take the vaccine since it was on the advice of health professionals. Moreover, 18 (2.9%) participants were unwilling to take the vaccine fearing that the vaccine caused COVID-19. Providing health education using various types of media could change these misconceptions.

According to the results of this study, people in the age group of 18–29 years were more hesitant to accept the vaccine than those aged above 29 years. This finding was in agreement with other research (38, 39). There were statistically significant associations between age and vaccine acceptance (15). These studies and our findings revealed that the intention to be vaccinated was greatest in the oldest age group. As comorbidities are more common among elderly people, as is the risk of complications due to COVID-19, it was expected that older people preferred vaccination when compared to the younger cohort. However, if they are not vaccinated for COVID-19, young people may serve as a source of infection to others and thus spread the disease. Thus, it is important educating young people on the importance of taking COVID-vaccines in order to improve vaccine acceptance rates among them and to control the epidemic.

Our results showed a high level of good knowledge of the COVID-19 vaccine among adults in Hawassa. Also, having low knowledge of the COVID-19 vaccine increased vaccine hesitancy. Similarly, studies from other settings and Ethiopia showed that knowledge scale is associated with COVID-19 vaccine acceptance or hesitancy (30, 40). Moreover, a systemic review paper confirmed that the likelihood of COVID-19 vaccine acceptance was higher among participants who had good knowledge of the vaccine (41). These findings inform us that improving COVID-19-related knowledge in the public could help in increasing vaccine acceptance. Thus, we suggest the importance of providing education about the vaccine using different modes of health education.

A high proportion of the study's participants had a positive attitude toward the COVID-19 vaccine and the attitude toward the COVID-19 vaccine predicted COVID-19 vaccine hesitancy. Other studies also reported an increased likelihood of COVID-19 vaccine acceptance among participants with positive attitudes toward the vaccine (14, 41). A positive attitude was among the determinant factors of COVID-19 vaccine acceptance in Ethiopia (30). Interventions to modify the attitude of people toward the COVID-19 vaccine could help in improving COVID-19 vaccination acceptance.

The likelihood of COVID-19 vaccine acceptance was higher among participants who had a history of chronic disease (41). Our results did not show such an association. This could be due to a bias introduced during measurement. Having chronic diseases was only measured by a question during the interview. We did not investigate or apply any other specific approach to confirm the existence of chronic diseases among the study participants. However, though the 95% CI was wide, having contracted COVID-19 disease showed a significant association with vaccine acceptance among our study population. People with a history of COVID-19 disease were more hesitant to take the vaccine than people without. The report by Albahri AH et al. did not show a statistically significant association between having COVID-19 with vaccine acceptance (15). In contrast to this, in Sudan, a history of COVID-19 illness increased the chance of vaccine acceptance among medical students (42). This could be because the participants involved in the study were medical students.

Studies reported the presence of statistically significant associations between gender and vaccine acceptance (7, 15, 30). Another study observed that being male was among the predictors of vaccine acceptance (19). This result could be due to an increased risk perception of the disease among men than among women. However, the association between gender and acceptance of the COVID-19 vaccine was not statistically significant among adults in the current study. However, in our study, COVID-19 vaccine hesitancy was higher among Orthodox Christians (43). According to reports from other settings, religiosity appeared to have an impact on the decision to be inoculated with the vaccine (44, 45). Involving religious leaders, particularly Orthodox Christian leaders, may be helpful to improve vaccine acceptance among this group of the population.

Our results provided important knowledge on vaccine hesitancy and its associated factors among adults in Hawassa City Administration. The sample used in this study was representative of Hawassa City Administration since we selected from both urban and rural parts of the City Administration. However, the sample size used in the study seems not sufficient to measure some of the risk factors of the outcome characteristics. As displayed in Table 4, variables such as having had contracted COVID-19 and attitude assessment variables had wide CIs, which confirms the insufficiency of the sample size to estimate these characteristics. However, other variables, such as age, religion, and knowledge assessment, showed association with vaccine hesitancy had narrow CIs. One possible limitation in this study was a recall bias related to measuring childhood vaccination and COVID-19 cases' contact history, which might have caused exposure misclassification. The association between exposure and outcome measures was not confirmatory as the data generated in this study was by a cross-sectional study design. There could also have been social desirability bias in measuring variables, such as having a family history of COVID-19, relatives and friends' history of COVID-19 sickness, and the characteristics used to measure COVID-19 vaccine knowledge. So we suggest readers take these limitations into consideration while interpreting our findings. Despite the limitations, the study is the first of its type conducted in the Sidama region, Ethiopia, which has approximately four million people. Our findings could be used in the region or other regions in Ethiopia.

## Conclusion

The majority of our study participants use mass media, had good knowledge of the COVID-19 vaccine, and had a positive attitude toward the COVID-19 vaccine. Compared to other reports, the COVID-19 vaccine hesitancy rate was lower among our study participants. The primary reason for willingness and non-willingness of taking the vaccine was to protect oneself and the notion that the vaccine caused COVID-19, respectively. Health professionals were the primary source of information about the vaccine. Age, religion, history of COVID-19 infection, knowledge related to the COVID-19 vaccine, and attitude toward the COVID-19 vaccine were factors that predicted vaccine hesitancy among the study participants.

Vaccine hesitancy is a complex phenomenon and there is no definitive evidence available regarding specific effective interventions to address it (46). It should be noted the pivotal role that healthcare providers and scientific journals as sources of information have in creating a positive impact on vaccination attitudes and uptake (47). In light of this and our analysis, we have suggested interventions to be applied that can affect vaccine hesitancy of adults in the study area. Providing health education on the importance of taking the COVID-19 vaccine is important, especially for people with the identified risk factors. Educating young people to receive the COVID-19 vaccine is important in order to improve the vaccine acceptance rate among them and to control the epidemic. This could improve their knowledge of the vaccine and prevent them from being the source of infection to others. Interventions to modify attitudes toward the COVID-19 vaccine could help in improving vaccination acceptance. This could be achieved by publicizing and sharing the experience of people who received the vaccine. Moreover, involving religious leaders could be helpful to improve the vaccine acceptance rate in the study area. As the majority of the study participants use mass media, using this type of media to deliver health education may be preferable.

## Data availability statement

The raw data supporting the conclusions of this article will be made available by the authors, without undue reservation.

## Ethics statement

The studies involving human participants were reviewed and approved by Institutional Review Board of College of Medicine and Health Sciences, Hawassa University. Written informed consent for participation was not required for this study in accordance with the national legislation and the institutional requirements.

## Author contributions

SY, AA, YW, TT, DD, MB, and EW conceived the study and wrote the manuscript. YW, TT, DD, and MB involved in data collection and writing the manuscript. EW critically reviewed the manuscript. All authors read and approved the final version of the manuscript.

## References

[1] WHO. Coronavirus disease (COVID-19) Situation Report – 153. Geneva: WHO (2020).

[2] WHO. COVID-19 advice for the public: Getting vaccinated. World Health Organization. Available online at: https://www.who.int/emergencies/diseases/novel-coronavirus-2019/covid-19-vaccines/advice (accessed July 14, 2021).

[3] Cui J Li F Shi ZL. Origin and evolution of pathogenic corona viruses. Nat Rev Microbial. (2019) 17:181–92. 10.1038/s41579-018-0118-9 30531947 PMC7097006

[4] Regulatory Affairs Professionals Society. COVID-19 vaccine tracker. Available online at: https://www.raps.org/news-and-articles/news-articles/2020/3/covid-19-vaccine-tracker (accessed October 1, 2021).

[5] Valderas JM Starfield B Sibbald B Salisbury C Roland M. Defining comorbidity: implications for understanding health and health services. Ann Fam Med. (2009) 7:357–63. 10.1370/afm.983 19597174 PMC2713155

[6] Anaki D Sergay J. Predicting health behavior in response to the coronavirus disease (COVID-19): worldwide survey results from early March 2020. PLoS ONE. (2021) 16:e0244534. 10.1371/journal.pone.0244534 33411827 PMC7790278

[7] Lazarus JV Ratzan SC Palayew A. A global survey of potential acceptance of a COVID-19 vaccine. Nat Med. (2020). 10.1101/2020.08.23.20180307 33082575 PMC7573523

[8] WHO. Coronavirus disease 2019 (COVID-19): situation report. Available online at: https://www.who.int/emergencies/diseases/novel-coronavirus-2019/situation-reports (accessed May 19, 2021).

[9] First case of COVID-19 confirmed in Ethiopia. WHO Africa. (2022). Available online at: https://www.afro.who.int/news/first-case-covid-19-confirmed-ethiopia

[10] FMOH. COVID-19) Ethiopia COVID-19 monitoring platform. Available online at: https://www.moh.gov.et/ejcc/en/node/196 (accessed December 06, 2022).

[11] Zikargae MH. COVID-19 in Ethiopia: assessment of How the Ethiopian Government 329 has executed administrative actions and managed risk communications and community 330 engagement. Risk Manag Healthc Policy. (2020) 13:2803. 10.2147/RMHP.S278234 33299368 PMC7721303

[12] Salam M. COVID-19 vaccine hesitancy worldwide: a concise systematic review of vaccine acceptance rates. Vaccines. (2021) 9:160. 10.3390/vaccines9020160 33669441 PMC7920465

[13] Xu Y Xu D Luo L Ma F Wang P Li H. A cross-sectional survey on COVID-19 vaccine hesitancy among parents from Shandong vs. Zhejiang. Public Health. (2021) 9:779720. 10.3389/fpubh.2021.779720 34805084 PMC8602062

[14] Magadmi RM Kamel FO. Beliefs and barriers associated with COVID-19 vaccination among the general population in Saudi Arabi. BMC Public Health. (2021) 21:1438. 10.1186/s12889-021-11501-5 34289817 PMC8294288

[15] Albahri AH Alnaqbi SA Alshaali AO Alnaqbi SA Shahdoor SM. COVID-19 vaccine acceptance in a sample from the United Arab Emirates general adult population: a cross-sectional survey, 2020. Front Public Health. (2021) 26:614499. 10.3389/fpubh.2021.614499 34381748 PMC8350048

[16] Bianco BA Giorgia DP Silvia A Concetta PP Francesca L Italo FA. Parental COVID-19 vaccine hesitancy: a cross-sectional survey in Italy. Expert Rev Vacc. (2022) 21:23013. 10.1080/14760584.2022.2023013 34949136

[17] Mohammed R Nguse TM Habte BM Fentie AM Gebretekle GB. COVID-19 vaccine hesitancy among Ethiopian healthcare workers. PLoS ONE. (2021) 16:1125. 10.1371/journal.pone.0261125 34919597 PMC8682893

[18] Tadele AF. Knowledge and proportion of COVID-19 vaccination and associated factors among cancer patients attending public hospitals of Addis Ababa, Ethiopia, 2021: a multicenter study. Infect Drug Resist. (2021) 14:4865–76. 10.2147/IDR.S340324 34848979 PMC8627267

[19] Sahile AT Mulugeta B Hadush S Fikre EM. COVID-19 vaccine acceptance and its predictors among college students in Addis Ababa, Ethiopia, 2021: a cross-sectional survey. Patient Prefer Adherence. (2022) 16:255–63. 10.2147/PPA.S348132 35136350 PMC8817737

[20] Tsegaw BT Amogne FK Demisse TL. Coronavirus disease 2019 vaccine acceptance and perceived barriers among university students in northeast Ethiopia: a cross-sectional study. Clin Epidemiol Glob Health. (2021) 12:848. 10.1016/j.cegh.2021.100848.100848 34395948 PMC8351076

[21] Handebo S Wolde M Shitu K Kassie A. Determinant of intention to receive COVID-19 vaccine among school teachers in Gondar city, Northwest Ethiopia. PLoS ONE. (2021) 16:3499. 10.1371/journal.pone.0253499 34166399 PMC8224841

[22] Zeleke AM Bayeh GM. Knowledge, attitude and practice towards COVID-19 and associated factors among pregnant women at debark town northwest Ethiopia: an institutional-based cross-sectional study. W J Adv Sci Technol. (2022) 1:31. 10.53346/wjast.2022.1.1.0031

[23] Mose A Yeshaneh A. COVID-19 vaccine acceptance and its associated factors among pregnant women attending antenatal care clinic in southwest Ethiopia: institutional-based cross-sectional study. Int J Gen Med. (2021) 14:2385–95. 10.2147/IJGM.S314346 34135622 PMC8197585

[24] Mose A. Willingness to receive COVID-19 vaccine and its determinant factors among lactating mothers in Ethiopia: a cross-sectional study. Infect Drug Resist. (2021) 14:4249–59. 10.2147/IDR.S336486 34703251 PMC8523808

[25] Hailemariam S Mekonnen B Shifera N. Predictors of pregnant women's intention to vaccinate against coronavirus disease 2019: a facility-based cross-sectional study in southwest Ethiopia. SAGE Open Med. (2021) 9:8454. 10.1177/20503121211038454 34434555 PMC8381422

[26] Mose A Haile K Timerga A. COVID-19 vaccine hesitancy among medical and health science students attending Wolkite university in Ethiopia. PLoS ONE. (2022) 17:3081. 10.1371/journal.pone.0263081 35077504 PMC8789154

[27] Abebe H Shitu S Mose A. Understanding of COVID-19 vaccine knowledge, attitude, acceptance, and determinants of COVID-19 vaccine acceptance among adult population in Ethiopia. Infect Drug Resist. (2021) 14:2015–25. 10.2147/IDR.S312116 34103948 PMC8179743

[28] Mesele M. COVID-19 vaccination acceptance and its associated factors in Sodo Town, Wolaita Zone, Southern Ethiopia: cross-sectional study. Infect Drug Resist. (2021) 14:2361–7. 10.2147/IDR.S320771 34194232 PMC8238544

[29] Sahile AT GD GDG Mgutshini T. COVID-19 vaccine acceptance level in ethiopia: a systematic review and meta-analysis. Can J Infect Dis Med Microbiol. (2022) 5:2313367. 10.1155/2022/2313367 36061634 PMC9436617

[30] Mose A Wasie A Shitu S Haile K Timerga A Melis T. Determinants of COVID-19 vaccine acceptance in Ethiopia: a systematic review and meta-analysis. PLoS ONE. (2022) 17:e0269273. 10.1371/journal.pone.0269273 35657814 PMC9165773

[31] Amsalu B Guta A Seyoum Z Kassie N Sema A Dejene W. Practice of COVID-19 prevention measures and associated factors among residents of dire dawa city, Eastern Ethiopia: community-based study. J Multidiscip Healthc. (2021) 14:219–28. 10.2147/JMDH.S292409 33564238 PMC7866932

[32] Henwood BF Redline B Lahey J. Surveying tenants of permanent supportive housing in skid row about COVID-19. J Health Care Poor Underserved. (2020) 31:1587–94. 10.1353/hpu.2020.0120 33416740

[33] Desye B. Prevalence and Determinants of COVID-19 vaccine acceptance among healthcare workers: a systematic review. Front Public Health. (2022) 10:941206. 10.3389/fpubh.2022.941206 35968421 PMC9366855

[34] Lenjiso T Tesfaye B Michael A Esatu T Legessie Z. Hawassa city adminstration health department 2010–2012 GTP assessment report. Booklet. (2013) 3:9–65.

[35] Yanto TA Lugito NPH Hwei LRY Virliani C Octavius GS. Prevalence and determinants of COVID-19 vaccine acceptance in South East Asia: a systematic review and meta-analysis of 1,166,275 respondents. Trop Med Infect Dis. (2022) 7:361. 10.3390/tropicalmed7110361 36355903 PMC9696885

[36] Giuseppe GD Pelullo CP Polla GD Montemurro MV Napolitano F Pavia M. Surveying willingness toward SARS-CoV-2 vaccination of healthcare workers in Italy. Expert Rev Vaccines. (2021) 20:881–9. 10.1080/14760584.2021.1922081 33900148

[37] Bullock J Lane JE Shults FL. What causes COVID-19 vaccine hesitancy? Ignorance and the lack of bliss in the United Kingdom. Human Soc Sci Commun. (2022) 9:1092. 10.1057/s41599-022-01092-w

[38] Noushad M Nassani MZ Koppolu P Alsalhani AB Samran A Alqerban A. Predictors of COVID-19 vaccine intention among the saudi arabian population: a cross-sectional Survey. Vaccines. (2021) 9:892. 10.3390/vaccines9080892 34452017 PMC8402383

[39] Al-Mohaithef M Padhi BK. Determinants of COVID-19 vaccine acceptance in saudi arabia: a web-based national survey. J Multidiscip Healthc. (2020) 13:1657–63. 10.2147/JMDH.S276771 33262600 PMC7686470

[40] Hanna P Issa A Noujeim Z Hleyhel M Saleh N. Assessment of COVID-19 vaccines acceptance in the Lebanese population: a national cross-sectional study. J Pharmaceut Policy Pract. (2022) 15:403. 10.1186/s40545-021-00403-x 35016705 PMC8749113

[41] Mekonnena D Mengistua BA. COVID-19 vaccine acceptance and its associated factors in Ethiopia: a systematic review and meta-analysis. Clin Epidemi Glob Health. (2022) 14:101001. 10.1016/j.cegh.2022.101001 35284688 PMC8898789

[42] Raja SM Osman ME Musa AO Hussien AA Yusuf K. COVID-19 vaccine acceptance, hesitancy, and associated factors among medical students in Sudan. PLoS ONE. (2022) 17:e0266670. 10.1371/journal.pone.0266670 35390097 PMC8989287

[43] Osur JO Chengo R Muinga E Kemboi J Sidibe M Rarieya M. Determinants of COVID-19 vaccine behaviour intentions among the youth in Kenya: a cross-sectional study. Arch Public Health. (2022) 80:4. 10.1186/s13690-022-00904-4 35733196 PMC9217729

[44] Garcia LL Yap JFC. The role of religiosity in COVID-19 vaccine hesitancy. J Public Health. (2021) 3:fdab192. 10.1093/pubmed/fdab192 34080617 PMC8195070

[45] Trepanowski R Drazkowski D. Cross-national comparison of religion as a predictor of COVID-19 vaccination rates. J Relig Health. (2022) 61:2198–211. 10.1007/s10943-022-01569-7 35556198 PMC9095816

[46] Dubé E Gagnon D MacDonald NE. Strategies intended to address vaccine hesitancy: review of published reviews. Vaccine. (2015) 33:4191–41203. 10.1016/j.vaccine.2015.04.041 25896385

[47] Jarrett C Wilson R O'Leary M Eckersberger E Larson HJ. Strategies for addressing vaccine hesitancy—A systematic review. Vaccine. (2015) 33:4180–90. 10.1016/j.vaccine.2015.04.040 25896377

